# A chickpea MAGIC population to dissect the genetics of complex traits

**DOI:** 10.1002/tpg2.70096

**Published:** 2025-08-24

**Authors:** Oluwaseun J. Akinlade, Hannah Robinson, Yichen Kang, Mahendar Thudi, Srinivasan Samineni, Pooran Gaur, Millicent R. Smith, Kai P. Voss‐Fels, Roy Costilla, Rajeev K. Varshney, Eric Dinglasan, Lee T. Hickey

**Affiliations:** ^1^ Queensland Alliance for Agriculture and Food Innovation The University of Queensland Brisbane Queensland Australia; ^2^ College of Agriculture, Family Sciences and Technology Fort Valley State University Fort Valley Georgia USA; ^3^ International Crops Research Institute for the Semi‐Arid Tropics (ICRISAT) Patancheru Telangana India; ^4^ Crop Diversification and Genetics, International Center for Biosaline Agriculture (ICBA) Dubai UAE; ^5^ School of Agriculture and Food Sustainability, Faculty of Science The University of Queensland Gatton Queensland Australia; ^6^ Department of Grapevine Breeding Hochschule Geisenheim University Geisenheim Germany; ^7^ Cawthron Institute Nelson New Zealand; ^8^ State Agricultural Biotechnology Centre, Centre for Crop and Food Innovation, Food Futures Institute Murdoch University Murdoch Western Australia Australia

## Abstract

Multiparent populations are now widespread in crop genetic studies as they capture more genetic diversity and offer high statistical power for detecting quantitative trait loci (QTLs). To confirm the suitability of using a recently developed chickpea (*Cicer arietinum* L.) multi‐parent advanced generation intercross (MAGIC) population for genetic studies, we characterized the diversity of the eight founder lines and explored the linkage disequilibrium decay, marker coverage, segregation distortion, allelic variation, and structure of the population. The MAGIC population was genotyped using whole‐genome sequencing; following marker curation, a total of 4255 high‐quality polymorphic single nucleotide polymorphism markers were used for genomic analyses. To demonstrate the effectiveness of the MAGIC population to dissect the genetics of key agronomic traits (days to 50% flowering and plant height), we employed both a genome‐wide mapping approach using fixed and random model circulating probability unification and a haplotype‐based mapping using the local genomic estimated breeding value approach. Our analyses confirmed the role of genomic regions previously reported in the literature and identified several new QTLs for days to 50% flowering and plant height. We also showed the potential for trait improvement through stacking the top 10 haploblocks to develop early flowering chickpea and selection of desirable haplotypes on chromosome 4 to improve plant height. Our results demonstrate the chickpea MAGIC population is a valuable resource for researchers and pre‐breeders to study the genetic architecture of complex traits and allelic variation to accelerate crop improvement in chickpea.

## Core Ideas

- Eight‐parent chickpea MAGIC population (1135 lines) offers intermediate level of diversity for genetic studies and breeding.
- Genome‐wide association study identified seven novel quantitative trait loci (QTLs) for flowering time and plant height with a strong enrichment of stress‐responsive, developmental, and regulatory genes.
- Haplotype mapping revealed key blocks, with stacking strategies for optimizing flowering and plant height.
- Rapid linkage disequilibrium decay (∼1 Mbp) in the multi‐parent advanced generation intercross (MAGIC) population supports mapping resolution suitable for MAGIC populations for mapping complex quantitative traits.
- Co‐located QTLs for flowering and height suggest pleiotropy, enabling simultaneous trait improvement.

## Plain Language Summary

Chickpea is a vital global crop, providing nutrition and supporting sustainable agriculture. To improve its resilience and productivity, understanding the genetic factors influencing complex traits is essential. We used a novel multi‐parent advanced generation intercross (MAGIC) population to study chickpea genetics. Our research identified several regions in the chickpea genome influencing flowering time and plant height, including seven previously unknown regions. By analyzing groups of markers (haplotypes), we demonstrated how combining optimal genetic variants can produce chickpeas that flower earlier or grow taller, depending on breeding goals. These findings provide new tools for breeders to develop chickpea varieties capable of withstanding environmental challenges and increasing yields. Furthermore, the MAGIC population is a valuable resource for exploring other complex traits, contributing to efforts to ensure global food security.

**Abbreviations**
DTF: days to 50% flowering; GEBV, genomic estimated breeding values;
GWAS: genome‐wide association study
LD: linkage disequilibrium
MAGIC: multi‐parent advanced generation intercross
PHT: plant height
QTL: quantitative trait locus
RIL: recombinant inbred line
SNP: single nucleotide polymorphism
SSD: single seed descent

## 1 INTRODUCTION

Chickpea (*Cicer arietinum* L.) is an important legume that is rich in nutrients and is a source of proteins, amino acids, and essential vitamins (Bar‐El Dadon et al., 2017; Paul et al., 2018). It is the second most consumed and third most‐produced legume globally (Upadhyaya et al., 2016; Varshney et al., 2013). Chickpea is a self‐pollinating, diploid (2*n* = 2*x* = 16) (van der Maesen, 1987) with a genome size of ∼738 Mb. Chickpea has two ecotypes: Desi, which has a small seed size with a thick seed coat, compared to Kabuli, which has a larger seed size with a smooth seed coat. Despite the growing importance of chickpea globally for food systems, current and future production is threatened by climate change (Akinlade et al., 2022). Consequently, there is an urgent need to improve the understanding of the genetic architecture of adaptive traits that can be utilized to develop more resilient, productive chickpea varieties.

Genetic gain in crop improvement programs relies heavily on genetic diversity (H. Li, Rasheed et al., 2018; Y. Li, Ruperao et al., 2018). The availability of reference genome sequences for chickpea has enhanced our understanding of the genetic architecture of key traits (Varshney et al., 2013, 2019). Allelic variation for traits is important for crop improvement and has been reported in many studies (Jha et al., 2021; Sannemann et al., 2018). Identifying genes and mapping specific QTLs influencing phenotypes or traits of interest requires an experimental population with broader genetic variability for the target traits (Ladejobi et al., 2016). Studies to date have focused primarily on bi‐parental mapping populations and diversity panels for QTL discovery (Kale et al., 2015; Nguyen et al., 2022; Paul et al., 2018; Pushpavalli et al., 2015; Sivasakthi et al., 2018; Thudi et al., 2014).

Multi‐parental mapping populations, such as multi‐parent advanced generation intercross (MAGIC) and nested association mapping, enable the evaluation of multiple alleles and greater recombination events to improve precision in QTL mapping (Ladejobi et al., 2016; Scott et al., 2020). The concept of the MAGIC population is a modification and extension of advanced intercross lines proposed by Darvasi and Soller (1995). In comparison to diversity panels, MAGIC populations can be advantageous as allelic variation within a set of founder lines can be reshuffled to improve mapping accuracy and balanced allele frequency can enhance statistical power (Ren et al., 2020). In practice, the intercrossing of founder lines breaks down the population structure via abundant recombination events (Han et al., 2020) and increases linkage disequilibrium (LD) decay (Rockman & Kruglyak, 2008).

MAGIC population development follows a straightforward design depending on the number of founder lines in the design (Stadlmeier et al., 2018), and populations have previously been developed in many crops including wheat (Huang et al., 2012), rice (Bandillo et al., 2013), maize (Holland, 2015), barley (Hemshrot et al., 2019), cowpea (Ravelombola et al., 2021), and soybean (Hashemi et al., 2022) and successfully used to gain new insight into important traits.

In this study, we aimed to explore the genetic architecture of a recently developed chickpea MAGIC population that contains 1135 lines derived from eight founders (Samineni et al., 2021; Thudi et al., 2024). The objectives were (1) to investigate the genetic architecture of the chickpea MAGIC population including population structure, LD, and linkage decay, and (2) to evaluate the utility of the chickpea MAGIC population for complex trait dissection using a case study for two agronomic traits: days to 50% flowering (DTF) and plant height (PHT).

## 2 MATERIALS AND METHODS

### 2.1 MAGIC population development

The population was developed over a 4‐year period (2009–2013) by the International Crops Research Institute of the Semi‐Arid Tropics, in partnership with the Indian Agricultural Research Institute (Samineni et al., 2021). Briefly, the eight founders were intercrossed using a stepwise approach. First, 28 two‐way crosses were performed, which were then used to generate 14 four‐way crosses, and finally used to generate seven eight‐way crosses. Following crossing, eight selfing generations were then performed using single seed descent (SSD) to generate inbred lines, and seed produced in the final generation was bulked to generate 1135 MAGIC lines examined in this study. A graphical representation of the crossing design and population development is presented in Figure S1.

**Core Ideas**

- Eight‐parent chickpea MAGIC population (1135 lines) offers intermediate level of diversity for genetic studies and breeding.
- Genome‐wide association study identified seven novel quantitative trait loci (QTLs) for flowering time and plant height with a strong enrichment of stress‐responsive, developmental, and regulatory genes.
- Haplotype mapping revealed key blocks, with stacking strategies for optimizing flowering and plant height.
- Rapid linkage disequilibrium decay (∼1 Mbp) in the multi‐parent advanced generation intercross (MAGIC) population supports mapping resolution suitable for MAGIC populations for mapping complex quantitative traits.
- Co‐located QTLs for flowering and height suggest pleiotropy, enabling simultaneous trait improvement.

### 2.2 Genome sequencing and single nucleotide polymorphism calling

This population was genotyped using whole‐genome sequencing method. Leaf tissue from 15‐day‐old seedlings was used for high‐throughput mini‐DNA extraction (Cuc et al., 2008). As described by Varshney et al. (2017), 500 bp insert sizes were constructed for all samples for the whole genome sequencing of the MAGIC population (Thudi et al., 2024). Illumina HiSeq2500 sequenced each library's paired‐end (PE) readings, and line data were trimmed and purified. Reads containing greater than 5% bases or polyA structures, as well as low‐quality reads, adapter contamination, and small insert size reads were filtered out. Additionally, read endpoint base quality was filtered out. Thereafter, the data were aligned on the Crop Development Centre Frontier genome (Varshney et al., 2013) using BWA (Burrows‐Wheeler aligner; H. Li et al., 2009). Post‐alignment processing was performed using Samtools, including sorting, marking duplicates, and indexing of BAM (binary alignment map) files (H. Li et al. 2009) and variant discovery and genotype calling were conducted through the Genome Analysis ToolKit (GATK v2.). The variant calling pipeline is referred as the “GATK Best Practices” workflow (DePristo et al., 2011). On average, the founders were sequenced to a depth of 48.74x, with individual depths ranging from 43.61x to 51.58x. For the MAGIC recombinant inbred lines (RILs), the average sequencing depth across the MAGIC lines was 6.97x, with a range from 4.45x to 14.56x.

### 2.3 Genotypic data quality control and imputation

VCF (variant call format) files generated for each chromosome, containing genotypic information from the MAGIC lines and founders, were merged using BCFtools (Danecek et al., 2021), resulting in a total of 494,325 single nucleotide polymorphisms (SNPs). The following quality control thresholds were used to remove SNP markers: minor allele frequency (MAF) ≤ 0.01, genomic read depth < 5, missing rate per SNP > 50%, and missing rate per sample > 50%. Monomorphic SNPs were removed from the dataset resulting in 29,391 SNPs. We used EAGLE v2.4.1 (Loh et al., 2016) for phasing and Minimac3 v2.0.1 (Howie et al., 2012) to impute missing genotypes. The sequence data of the eight founders were used as the reference to impute the genotypes of the 1135 MAGIC lines (target sample). The accuracy of the imputation was calculated using Minimac's *R* ^2^ statistic (*R* ^2^). Only SNPs with *R* ^2 ^> 0.95 were kept for downstream analysis. Finally, to reduce marker redundancy, highly correlated markers (>0.99) were removed resulting in 4255 high‐quality SNP markers for the subsequent analysis.

### 2.4 Investigating diversity of founders

The eight founders used for the development of this MAGIC population were a combination of released cultivars and elite breeding lines preferred across Africa and Asia (Table S1).

To investigate the genetic diversity represented by the founders, the eight genotypes (founders) were co‐analyzed with a diversity panel comprising 91 desi genotypes collected across the world from 1970 to 2002. A common set of 2286 polymorphic SNPs across all 99 genotypes was used for analysis. First, Roger's genetic distance was calculated using the R package “SelectionTools” version 22.1 (http://population‐genetics.uni‐giessen.de/software/). Then, based on the distance matrix, *k*‐means clustering was performed, whereby the optimum *k* number was determined using the R package “NbClust” version 3 (Charrad et al., 2014). We constructed a dendrogram of relatedness using complete clustering and heatmap to show where the eight founders sit within the diversity panel.

### 2.5 Population structure and genetic analysis among the founders and MAGIC lines

To analyze the population structure, the genotypic data of the 1135 MAGIC lines and the eight founder lines were merged using PLINK (Purcell et al., 2007). The merged genotypic data, comprised of 4255 high‐quality SNP markers, were used to calculate Roger's genetic distance. Based on the distance matrix, a classical multidimensional scaling was performed. To visualize the population structure, the first four principal components (PCs) were plotted. The MAGIC population was grouped based on the seven pedigrees defined by crossing order.

The genetic structure was explored by estimating the LD decay for the entire MAGIC population. The pairwise coefficient of correlations (*r* ^2^) between adjacent SNP markers was calculated. The decay of LD in relation to the SNP position was determined by fitting the locally estimated scatterplot smoothing curves (Wickham & Sievert, 2016). SNP markers greater than 50 Mbp position were considered unlinked and eliminated from the analysis (Roncallo et al., 2021). Distributions of minor allele frequency and observed heterozygosity were also calculated (Figure S2). All analyses were conducted in R using SelectionTools package and visualized using gglplot.

### 2.6 Phenotyping days to 50% flowering and plant height

To evaluate the potential of the MAGIC population to map QTL and dissect the genetics of complex traits, we focused on two key agronomic traits: DTF and PHT (cm). The population was evaluated in the field in 2013 at the International Crops Research Institute for the Semi‐Arid Tropics. DTF is noted visually when 50% of the plants within the plot are flowering, and PHT (cm) is measured with ruler at maturity. The experimental design was augmented with parents as checks with eight replicates for estimation of error variance, as described by Samineni et al. (2021). The MAGIC lines were not replicated in this augmented design. Prior to the genome‐wide association mapping analysis, phenotypes were adjusted for block effect, and error variance was estimated using founder lines as checks in the experimental design. We used the ASReml‐R package (Butler et al., 2009) in R to fit the following mixed model for the 2 years (R Core Team, 2021):

y=Xβ+pw+zg+∈,

where ***y*** represents a matrix of phenotypic values for each trait per year, *β* represents a vector of block effect, ***w*** represents a vector of the parents of the MAGIC lines, **g** represents the vector of MAGIC lines, and ∈ is error variance. For the base model, we fitted the parents (checks) as fixed effect, and the MAGIC lines as random. We removed outliers after running the base model and tested for significance of both the block and row effects in a fixed and random forms using the Wald test statistics and loglikelihood ratio, respectively. Thereafter, the final model was the same as the base model having confirmed that block and row effects were not significant in fixed and random effects. The adjusted phenotypic data were used for the downstream genome‐wide association analysis.

### 2.7 Genome‐wide association studies

Genome‐wide association studies (GWASs) used 4255 SNP markers and phenotypes for DTF and PHT. GWAS was conducted in R using genomic association and prediction integrated version 3 (Wang & Zhang, 2021) following the statistical computations of the fixed and random model circulating probability unification as described by Liu et al. (2016). Marker‐trait associations for DTF and PHT were subjected to multiple testing corrections to identify significant QTL (Gao et al., 2008). We used quantile‐quantile plots that represented expected and observed probability of obtaining associations of markers with respective traits −log10(*p*) values and a cut‐off threshold of −log10(*p*) ≥ 4.0 to reduce the type II error rate. Significant SNP markers physically linked in LD (*r* ^2^ ≥ 0.5) were considered as a single QTL. Significant QTLs were compared to known QTLs in the literature.

### 2.8 Haplotype‐based mapping

#### 2.8.1 Construction of LD blocks

Genome‐wide LD block construction was performed using an algorithm in the R package “SelectionTools”. First, *r* ^2^ values across SNP markers were calculated throughout each chromosome. An LD threshold of *r* ^2^ = 0.5 was applied to group SNP markers. A genome scan was performed where flanking markers within the threshold were grouped together as an LD block. To accommodate for incorrectly positioned markers or biased LD estimations, each block had a marker tolerance parameter of *t* = 3. This meant that even if a flanking marker did not match the LD grouping requirement, the block was still extended if at least *t* neighboring flanking markers did. If the *r* ^2^ value of a pair of markers was larger than the threshold value of 0.5, the pair was identified as a new LD block. Flanking markers on either side of the LD block were considered in the following phase and included in the same block if their pairwise *r* ^2^ values with the LD block's corresponding outside markers were also above the threshold value. The block was deemed complete if there were more than t flanking markers with a lower LD than *r* ^2^ = 0.5. This process was repeated until all the markers had been assigned to their appropriate blocks.

### 2.9 Estimation of local GEBV and block variance

The flowchart of the procedure is according to Voss‐Fels et al. (2019). First, we used the ridge‐regression best linear unbiased prediction model to estimate all marker effects simultaneously. Thereafter, we summed up the predicted allelic effects of each realized haplotype for all genome‐wide LD blocks, also referred as “local” genomic estimated breeding value (local GEBV). Finally, the variances of each LD block were estimated on all the local GEBVs for all the haplotypes on each LD block (Voss‐Fels et al., 2019). LD blocks with high variances were considered containing the QTLs associated with DTF and PHT (Brunner et al., 2024).

### 2.10 Co‐location analysis of QTL

The genomic regions detected in this study were positioned with the already reported QTL using the physical position and displayed using MapChart version 2.3 (Voorrips, 2002). We investigated the co‐location of the genomic regions associated with vigor and other agronomic traits already reported.

#### 2.10.1 Haplotype analyses and haplotype block stacking

In order to generate an estimate of the genealogies between common allelic variants for the most significant haploblock in our MAGIC population for DTF and PHT, we performed haplotype analysis. Haplotypes that were in strong LD were used to generate haplotype networks by employing the TCS (Templeton, Crandall, and Sing) phylogenetic network approach that was implemented in the software PopART (Leigh et al., 2015). The means of lines carrying haplotype variants were then fitted to a linear model and tested for statistical significance in R.

The impacts of stacking haplotypes at the 10 blocks with the highest variance were analyzed for both traits. For the “desirable” haplotypes, we considered the haplotypes with negative effects for DTF, as early‐maturing chickpea plant could be advantageous, particularly when grown in a terminal drought‐prone environment. While for the PHT, we considered the positive effects haplotypes (associated with taller PHT) of the block with highest variance as shown to be advantageous. Taller plants height has a higher yield potential and enhanced efficiency for mechanical harvesting.

## 3 RESULTS

### 3.1 Marker distribution, founder diversity, and genetic structure of the MAGIC population

**FIGURE 1:**
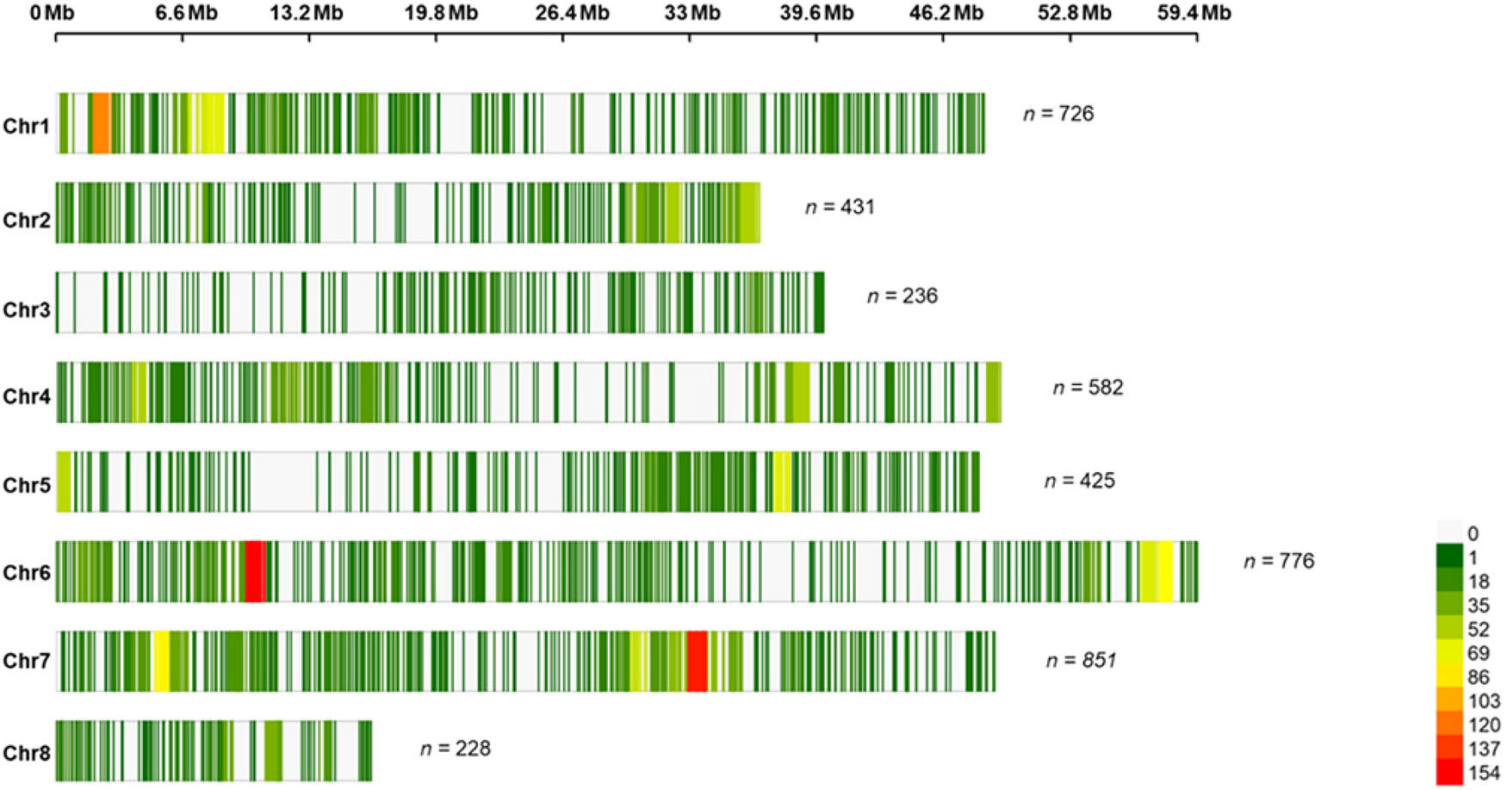
Single nucleotide polymorphism (SNP) density and distribution in the multi‐parent advanced generation intercross (MAGIC) population across the eight chromosomes of chickpea. The number of SNP markers is estimated and visualized in 1 Mb window size for each of the chromosomes. The number of SNPs per Mb is color‐coded with red, which indicates high SNP density, while green indicates low SNP density.

**FIGURE 2:**
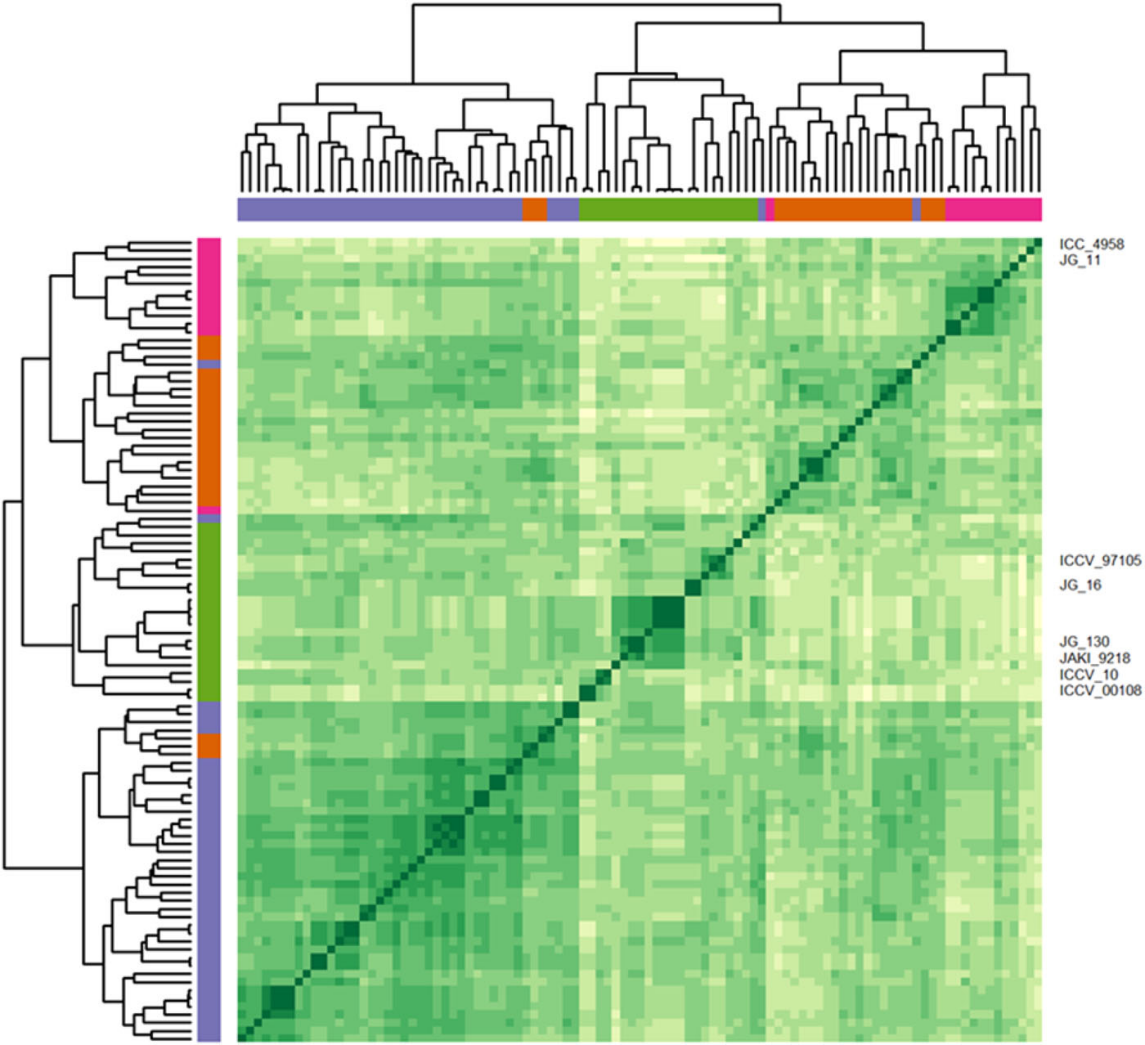
Genetic relationships among diversity panel (*n = *91) and the eight founder lines of the multi‐parent advanced generation intercross (MAGIC) population. Complete linkage hierarchical analysis and *k*‐means clustering with *k = *4 optimum number of clusters were identified: Cluster 1 (*n *= 41, purple), Cluster 2 (*n *= 13, pink), Cluster 3 (*n *= 22, green), and Cluster 4 (*n *= 22, orange). The eight founder lines are indicated on the right‐hand side of the heatmap.

Following the removal of highly correlated markers, 4255 SNPs remained. Markers varied and were unequally distributed across and within the eight chromosomes (Figure 1). Based on the physical map, chromosome 3 had the lowest coverage of markers (six markers/Mbp), whereas chromosome 7 showed the highest coverage (17 markers/Mbp) (Figure 1). Across all chromosomes, there were some regions with low marker coverage indicating areas of genotyping bias. Co‐analysis of the MAGIC founders with the diversity panel provided insights into the genetic variation represented in the eight founders. Complete linkage hierarchical clustering subdivided the MAGIC founders and the diversity panel into two major clusters and further subdivided them into four distinct groups according to *k*‐means clustering with the optimum *k* number (Figure 2). Clusters 1 (*n *= 41) and 4 (*n *= 23) consisted of majority of the diversity panels, while Clusters 2 (*n* = 13) and 3 (*n* = 21) comprised of mixture of diversity panels and the MAGIC founder lines. Founders ICC 4958 and JG 11 are genetically more similar (Cluster 2) and differentiated with ICCV 97105, JG 16, JD 130, JAKI 9218, ICCV 10, and ICCV 0108 (Cluster 3) (Figure 2). There were notable conserved chromosomal regions within the eight founders and high allelic variation as evident on chromosomes (Ca) 3, 5, and 6 (Figure S3).

**FIGURE 3:**
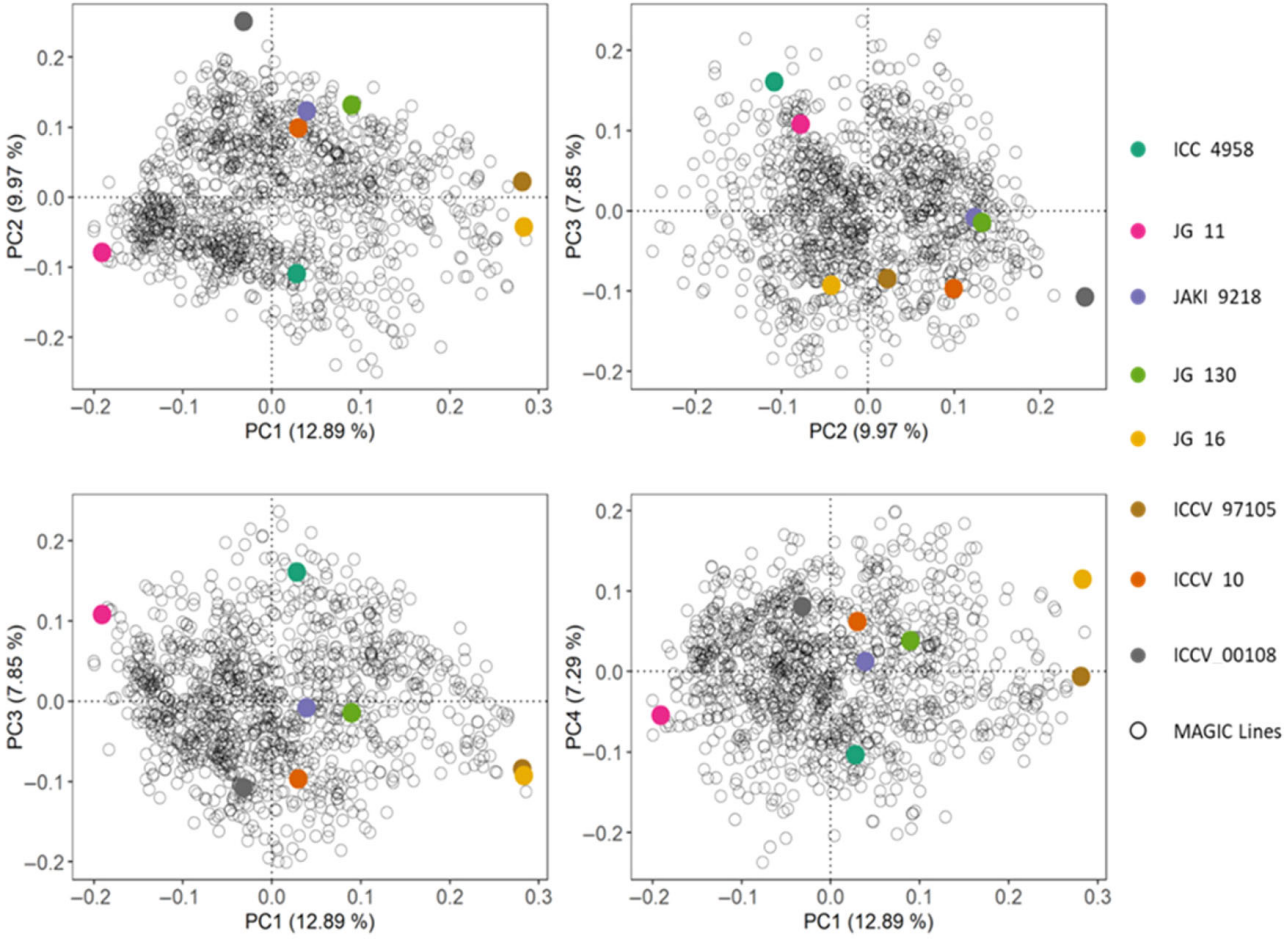
Population structure of the multi‐parent advanced generation intercross (MAGIC) population and the eight founder lines. Principal component analysis based on the pairwise (Roger's genetic distances) calculated using 4255 high‐quality polymorphic single nucleotide polymorphism (SNP) markers for 1135 MAGIC lines and 8 founder lines. The variation explained by principal components (PCs) was PC1 = 12.89%, PC2 = 9.97%, PC3 = 7.85%, and PC4 = 7.29%. Each founder is a distinct color, and the MAGIC lines are displayed in black.

**FIGURE 4:**
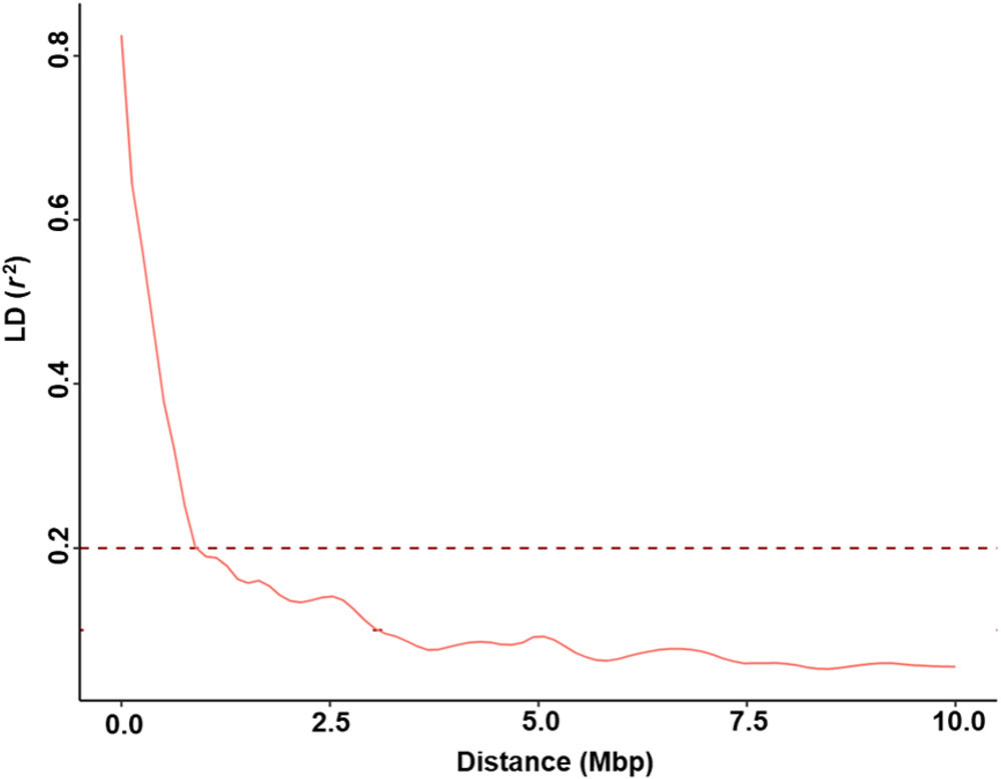
Genome‐wide linkage disequilibrium (LD) decay plot of the entire multi‐parent advanced generation intercross (MAGIC) population using 4255 high‐quality single nucleotide polymorphism (SNP) markers. Pairwise LD values (*r* ^2^) are plotted against physical SNP position. The red inner trend line is the locally estimated scatterplot smoothing (LOESS) curve. LD decay is shown as the intersection of the curves at the threshold of *r^2^* = 0.2 at the distance of ∼1 Mbp.

Intercrossing of the eight founders followed by selfing generations successfully reshuffled the genome and resulted in a MAGIC population with minimal population structure (Figure 3), and clustering of lines did not generally correspond with crossing order (Figure S4). Analysis of the whole genome SNP data for the entire MAGIC population revealed that PCs1–4, explained 12.89%, 9.97%, 7.85%, and 7.29% variance, respectively. The cumulative variation explained by the first two PCs accounted for 22.86% of the genetic variability in the population. The genetic distance showed limited population structure relative to the founder lines (Figure 3). LD within the MAGIC population showed a faster decay across the genome with a threshold of *r^2^* = 0.2 at a distance of ∼1 Mbp (Figure 4). Overall, the mean MAF and observed heterozygosity were 0.290 and 0.021, respectively (Table S3; Figure S2).

### 3.2 Phenotypic variation for plant height (PHT) and days to flowering (DTF) in the MAGIC population

**FIGURE 5:**
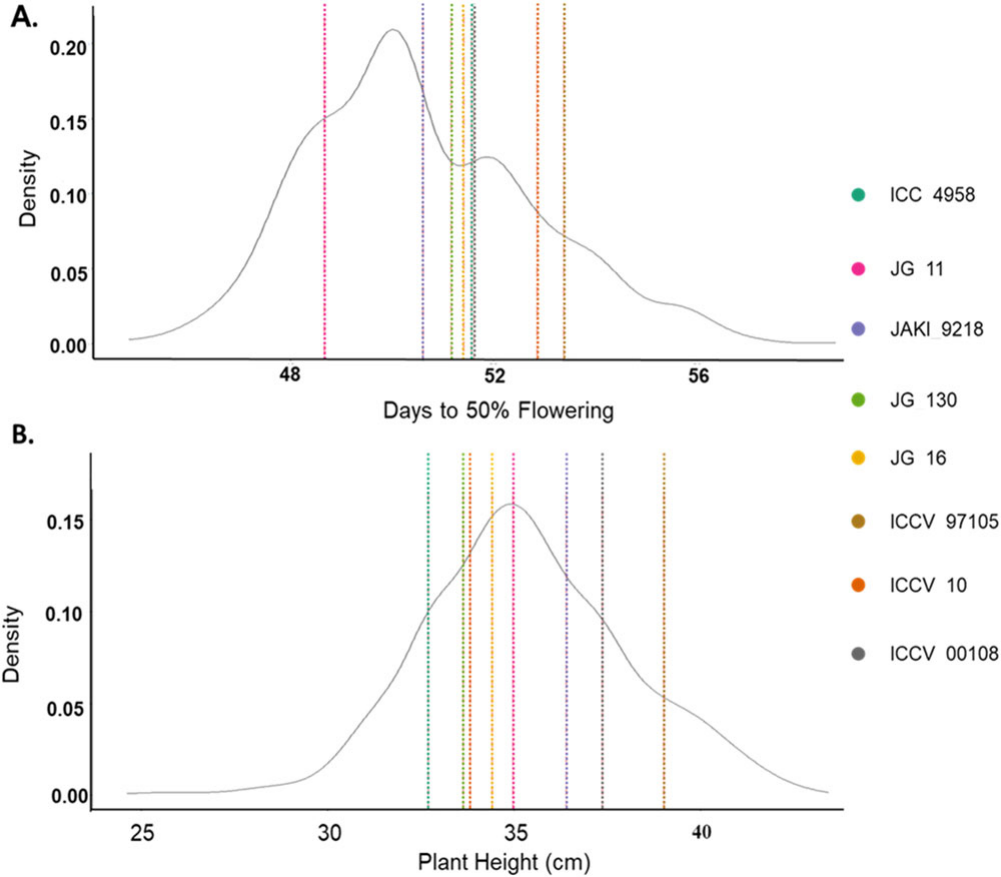
Phenotypic variation across multi‐parent advanced generation intercross (MAGIC) population grown under field conditions. (A) Density plot of days to 50% flowering showing the positions of the founders (each indicated in a distinct color). (B) Density plot of the MAGIC population for plant height, the distribution showing the positions of the founders (each indicated in a distinct color).

Evaluation of MAGIC lines in the field across two seasons revealed transgressive segregation for both PHT and DTF, as some of the MAGIC lines had higher or lower phenotypic values compared to the founders (Figure 5). For example, PHT for founders ranged from 32.70 (ICC 4958) to 39.01 cm (ICCV 97105), whereas MAGIC lines ranged from 24.63 to 43.42 cm (Figure 5), indicating that particular MAGIC lines are shorter or taller than the eight founders. For DTF, the founders reached 50% flowering between 48.67 days after sowing (JG 11) and 53.37 days after sowing (ICCV 97105), whereas MAGIC lines ranged from 44.83 to 58.71 days (Figure 5), demonstrating that progeny showed a wider range in flowering behavior than the founders. The overall correlation between DTF and PHT among the population was *r^2^* = 0.11 (*p* < 0.01).

### 3.3 Genomic regions associated with plant height and flowering

**FIGURE 6:**
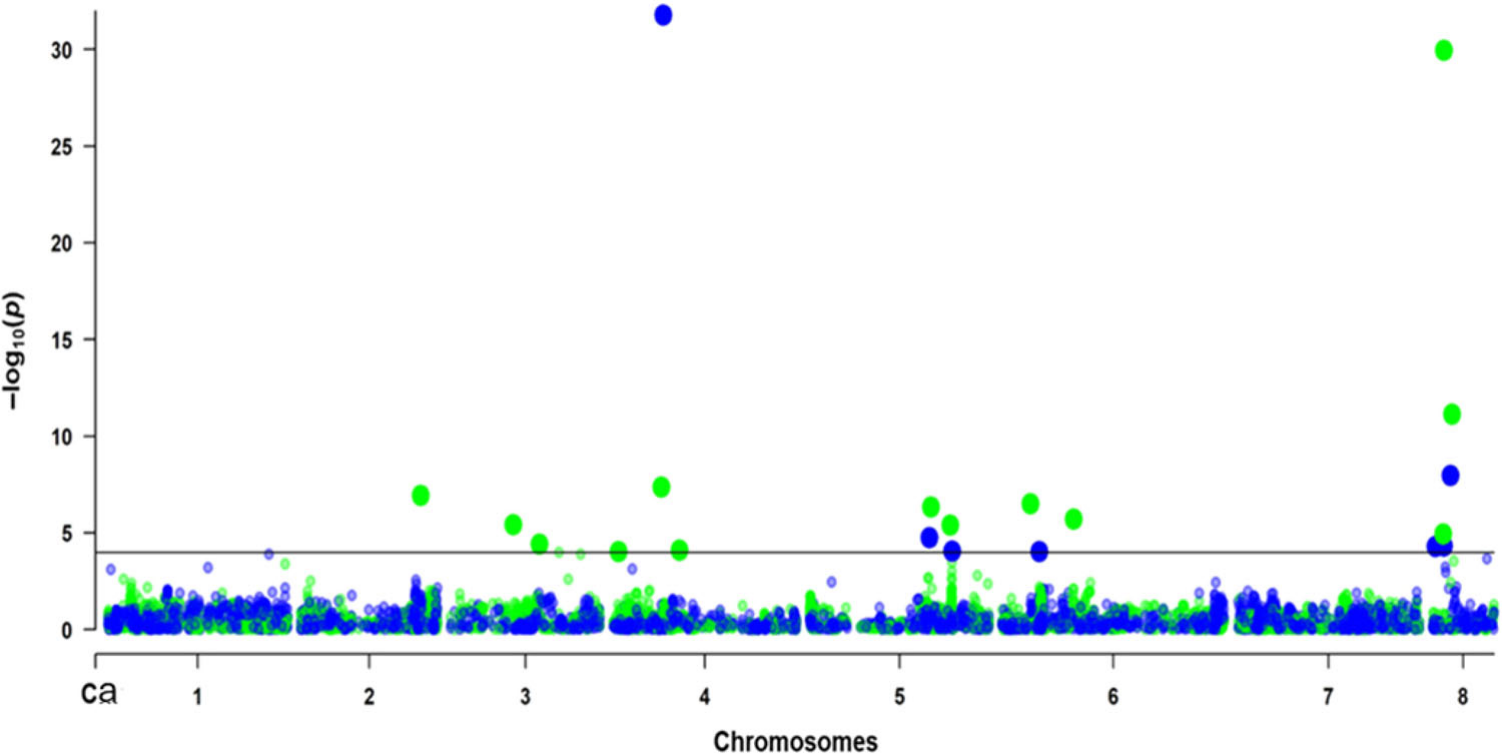
Manhattan plot illustrating results from genome‐wide association mapping in the multi‐parent advanced generation intercross (MAGIC) population for days to 50% flowering (green) and plant height (blue) using 1135 MAGIC lines and 4255 high‐quality single nucleotide polymorphism (SNP) markers. Each dot on the Manhattan plot represents a single SNP marker. The horizontal line represents the threshold for significant marker‐trait associations (−log_10_(*p*) = 4.00), which are displayed as a larger sized dot.

A total of 19 unique SNPs (−log10(*p*‐value) = 4.00) were associated with both PHT and DTF (Figure 6; Figure S5). Markers associated with PHT were detected on chromosomes 4, 5, 6, and 8, whereas markers associated with DTF were detected on chromosomes 2, 3, 4, 5, 6, and 8. The genome‐wide LD was employed as a criterion to identify whether two closely positioned associated markers were representing the same QTL region. SNP markers with LD ≥ 0.1 are linked (Table S2). A total of 12 QTL regions were identified based on this threshold (Table 1).

**TABLE 1:** Summary of the quantitative trait locus (QTL) for days to 50% flowering (Q‐DTF) and plant height (Q‐PH) in the chickpea multi‐parent advanced generation intercross (MAGIC) population.

*Q*/*N*	Chr	QTL	Marker.Position	DTF −log10(*p*)	PH −log10(*p*)	QTL	References
1	2	*Q‐DTF1*	M2.32058207	6.93	NA	Potentially novel	
2	3	*Q‐DTF2*	M3.16743467	5.43	NA	*qtlDtf‐3.1*	Abdi et al. (2016)
3	3	*Q‐DTF3*	M3.23724626	4.41	NA	Potentially novel	
4	4	*Q‐DTF4*	M4.1487970	4.03	NA	*qtlDtf‐4.1*	Abdi et al. (2016)
5	4	*Q‐DTF5*	M4.12926479	7.37	NA	*qtlDtf‐4.2*	Abdi et al. (2016)
	4	*Q‐PH1*	M4.13421126	NA	31.77	*qPH4.1/qtlDtf‐4.3*, *Ca4_Vqtl*	Kujur et al. (2016)
6	4	*Q‐DTF6*	M4.17785478	4.12	NA	*qtlDtf‐4.4*	Kujur et al. (2016)
7	5	*Q‐PH2*	M5.32027655	NA	4.77	Potentially novel	
8	5	*Q‐DTF7*	M5.32448226	6.34	NA	*qtlDtf‐5.1*	Kujur et al. (2016)
	5	*Q‐DTF8*	M5.37601329	5.41	NA	*qtlDtf‐5.2*	Kujur et al. (2016)
	5	*Q‐PH3*	M5.38102595	NA	4.07	Potentially novel	
9	6	*Q‐DTF9*	M6.7564965	6.5	NA	Potentially novel	
	6	*Q‐PH4*	M6.9942982	NA	4.04	*QR3pht01*	Gupta et al. (2015); Varshney et al. (2014)
10	6	*Q‐DTF10*	M6.19084539	5.73	NA	Potentially novel	
11	8	*Q‐PH5*	M8.673893	NA	4.3	*qPH8.1*	Kujur et al. (2016)
12	8	*Q‐DTF11*	M8.2762755	4.95	NA	*qtlDtf‐8.1*	Jaganathan et al. (2015); Varshney et al. (2014)
	8	*Q‐DTF12/Q‐PH6*	M8.3011407	29.96	4.33	*qtlDtf‐8.2*	Jaganathan et al. (2015); Varshney et al. (2014)
	8	*Q‐PH7*	M8.4764769	NA	7.96	*qPH8.1*	Jaganathan et al. (2015); Kujur et al. (2016); Varshney et al. (2014)
	8	*Q‐DTF13*	M8.5166666	11.15	NA	Potentially novel	

Abbreviations: LD, linkage disequilibrium. NA, not applicable.

A total of six unique QTLs were associated with DTF, and two unique QTLs associated with PHT. The most significant QTL for PHT (*Q‐PH1*) is closely linked with a QTL for DTF (*Q‐DTH5*). Interestingly, the most significant QTL for DTF (*Q‐PH12*) was also detected associated with a QTL for PHT (*Q‐PH6*) by a unique marker (M8.3011407). This corresponds to a genomic region previously identified and associated with DTF, and our results also identified this region to be associated with PHT. Notably, seven potentially novel QTLs for both PHT (*n *= 2) and DTF (*n *= 5) were identified using the MAGIC population. Overall, a total of four common closely linked QTLs were identified modulating both PHT and DTF.

### 3.4 Haplotype and effects of stacking of importance region for DTF and PHT

**FIGURE 7:**
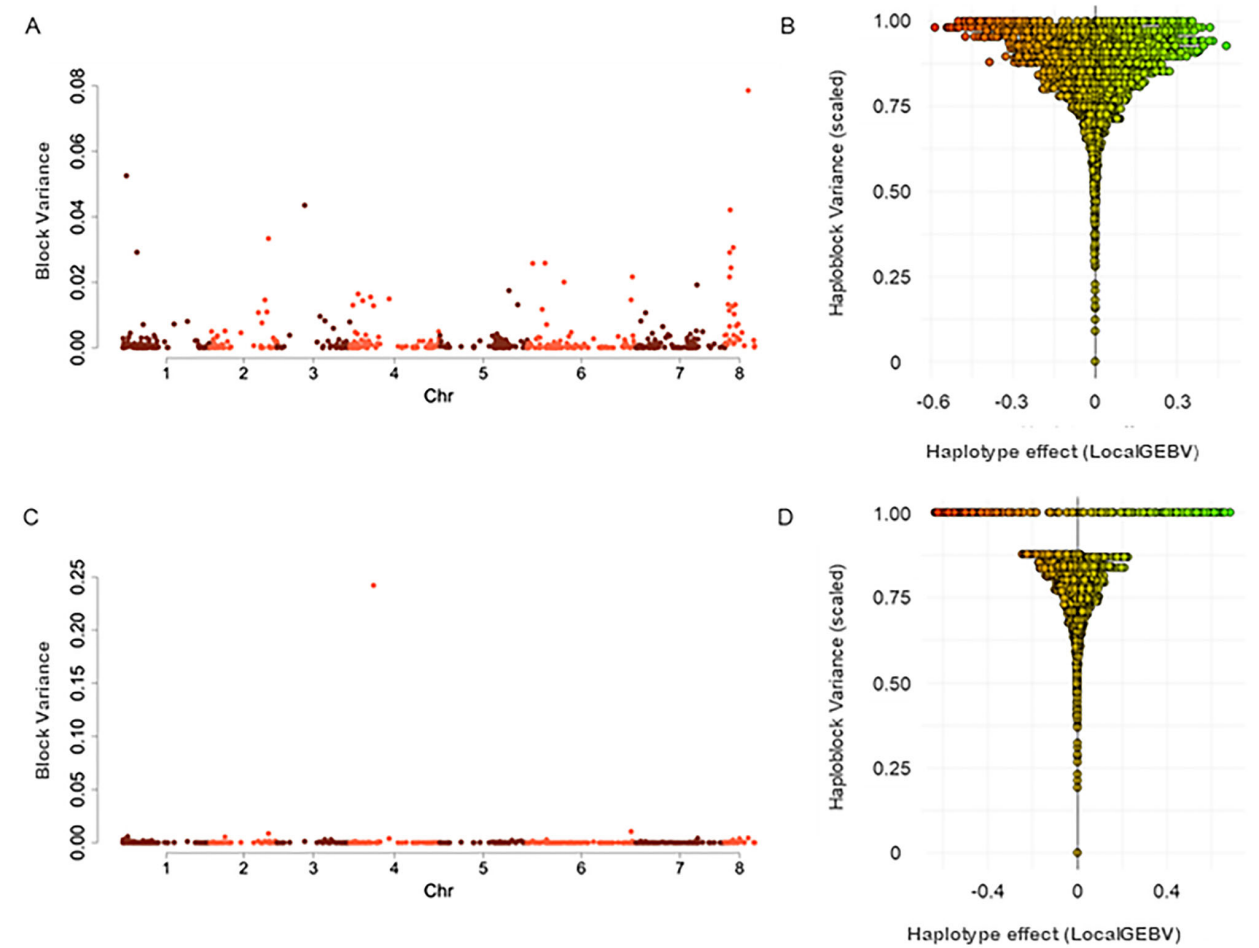
Haplotype‐based mapping of days to 50% flowering (DTF) and plant height (PHT) haplotype effects and variances. (A and C) Manhattan plot of haploblocks showing variances on the eight chickpea chromosomes, and (B and D) corresponding haplotype effects (local GEBV [genomic estimated breeding values]) in high variance blocks for DTF and PHT, respectively. For visualization, haploblock variance is scaled to clearly illustrate the relationship of haplotype effects and haploblock variance.

We also performed haplotype‐based mapping for DTF and PHT using the local genomic estimated breeding value approach (Table S4). We identified the top 10 haplotype blocks for both DTF and PHT based on the highest variances. LD block *b000646* on chromosome 8 showed the highest variance for DTF (Figure 7A), and *b000250* on chromosome 4 ranked highest for PHT (Figure 7C).

To explore the distribution of haplotype effects for DTF and PHT, Figure 7B (DTF) and D (PHT) shows their effects plotted against a scaled (0–1) variance for each block. Interestingly, both negative and positive effect haplotypes were evident for both traits (Figure 7B,D). For DTF, negative effect haplotypes were associated with earlier flowering, while for PHT, positive effect haplotypes were associated with taller PHT.

**FIGURE 8:**
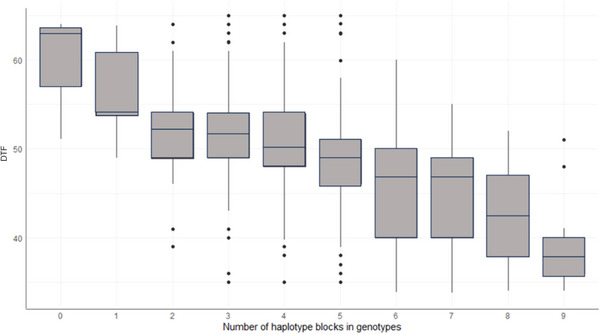
The effect of stacking top 10 haplotype blocks with negative effects (favorable haplotype blocks) on days to 50% flowering (DTF). Horizontal line within each boxplot represents the median value.

**FIGURE 9:**
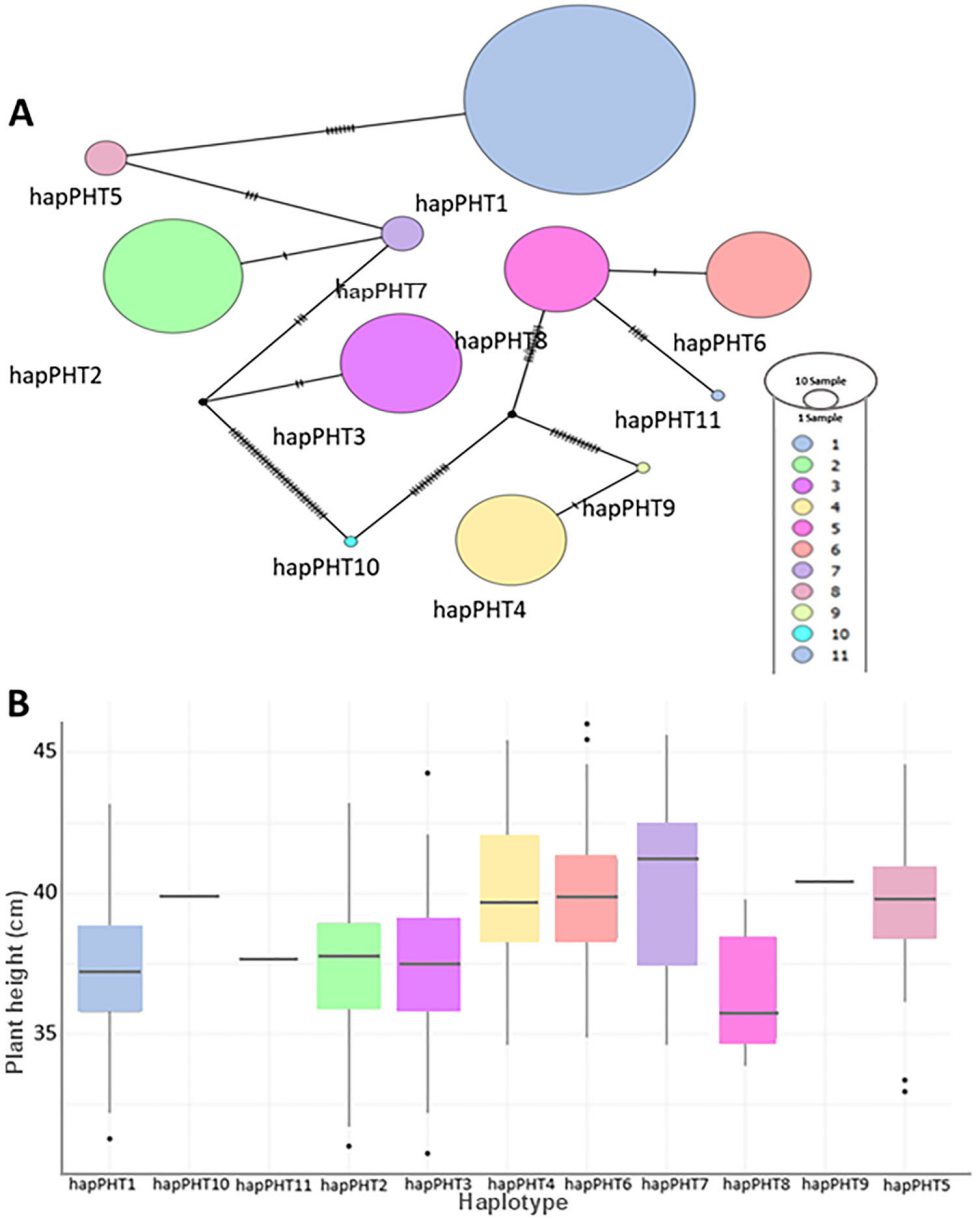
Haplotype (hap) analysis of *b000250*, which showed the highest variance for plant height. (A) Haplotype network of the 11 haplotype variants segregating in the multi‐parent advanced generation intercross (MAGIC) population. (B) Boxplot illustrating the plant height (PHT) phenotypes of lines carrying the haplotype variants. Middle horizontal line represents mean plant height.

Our in silico haplotype stacking analysis revealed a linear trend between the number of desirable haplotype blocks and DTF (Figure 8). Meanwhile, because the visualization of the block variance showed a distinct block with highest variance on chromosome 4 (Figure 7D), haplotype stacking analysis that was performed using this highest variance PHT block revealed a similar trend (Figure 9).

The block was on chromosome 4 and ranged from 11.84 to 14.18 Mbp. Haplotype analysis of the block revealed a total of 11 haplotype variants (hapPHT1–11) segregating in the MAGIC population (Figure 9). Of these, four showed high frequencies (greater than 70 individuals in the whole population), while four showed medium frequencies (more than 10 individuals), and three haplotypes were deemed rare (1 in the whole population). To understand the PHT response among haplotypes, we compared the haplotypes using the phenotypic dataset for their cumulative effects. HapPHT1 is most common haplotype with 338 counts in the population (∼32%), but hapPHT7, which was only present in 1.1% of the population, displayed the highest mean for PHT. The haplotypes hapPHT1, hapPHT2, and hapPHT3 performed similarly, and hapPHT4, haPHT5, and haPHT6 also performed similarly for PHT.

## 4 DISCUSSION

In this study, we analyzed the genetic architecture of a recently developed chickpea MAGIC population derived from eight genetically diverse founder lines and explored its utility for dissecting the genetics of traits to support crop improvement programs. Through the analysis of a panel of diverse chickpea accessions collected from 14 countries across the globe, we revealed the high degree of genetic diversity captured by MAGIC founder lines. As expected, the resulting population showed little to no population structure related to founder effect or crossing order and a high rate of LD decay, which are common features of MAGIC populations developed in other crop species. GWAS identified previously reported and novel QTL controlling both flowering and PHT, including four genomic regions that were associated with both traits. Haplotype analysis revealed new alleles combination that could potentially be used to breed improved chickpea varieties with earlier flowering to avoid terminal drought or taller PHT, which could improve efficiency of mechanized harvesting.

### 4.1 Founders exhibit the genetic diversity essential for MAGIC population development

Genetic diversity is an essential ingredient to support genetic gain in crop improvement programs. MAGIC populations derived from diverse founders provide useful resources to explore and reshuffle genetic diversity that could be integrated into a breeding program. The chickpea MAGIC population was generated from eight diverse Asian and African elite chickpea varieties. To explore the genetic variation represented by the eight founders, we studied a diverse panel of 91 chickpea genotypes. Cluster analysis using whole‐genome SNP markers determined that the MAGIC founders captured a substantial proportion of diversity present in the 91 desi varieties, with founders falling into two of the four clusters (Figure 2). Therefore, the degree of diversity captured in this MAGIC population makes it a valuable resource for pre‐breeders and plant scientists to study important traits for chickpea improvement compared to using biparental populations. This is because biparental populations capture allelic variation from only two parental lines, whereas the MAGIC population captures and reshuffles the genetic diversity of eight founders, thereby representing a broader and more diverse genetic base. Genome‐wide allele assessment of the founder lines revealed key chromosome regions that were polymorphic and conserved among select founders (Figure S3); however, as the crossing strategy recombined parental alleles from eight founders, the MAGIC progeny showed polymorphic SNP variation across most of the genome. Overall, genomic characterization of the founders showed they represent a degree of genetic diversity present in chickpea germplasm sourced from Asia and Africa.

### 4.2 A MAGIC resource with low population structure and rapid LD decay

It is critical to consider population structure, LD decay, and genetic relatedness as key features of a mapping population that can determine the success and outcome of genotype–phenotype association studies (Ladejobi et al., 2016; Scott et al., 2020). The intercrossing strategy employed to generate an eight‐way MAGIC population aims to recombine the genome of diverse founders in the resulting progeny. As a result, low population structure was observed. For example, only 12.89% and 9.97% of variation in the population was explained by the first two PCs (Figure 3). Despite the fact that founder lines were crossed in different orders to create the MAGIC progeny, no clear clustering pattern was associated with crossing order (Figure S4). Similar results have been reported for MAGIC populations in other crops including common bean (Diaz et al., 2020), cowpea (Huynh et al., 2018), and rice (Bandillo et al., 2013). This indicates that MAGIC progeny are truly genetic mosaics of the founders used and highlights the effectiveness of the approach across species to help dissect the genetic architecture of the trait of interest.

A key driver of LD decay during population development is recombination, where higher recombination rate leads to higher LD decay. However, several other factors can also influence LD decay including mutation rate, relatedness (kinship), selection, genetic diversity, and population structure (Bastien et al., 2014). In this chickpea MAGIC population, LD decayed rapidly and reached a threshold of *r* ^2^ = 0.2 at a distance of ∼1 Mbp. This is faster LD decay when compared to decay in chickpea biparental populations (Y. Li et al., 2017). The study by Thudi et al. (2024) analyzed the same MAGIC population, but used a different set of markers, and reported a similar threshold of ∼1.2 Mbp (Thudi et al., 2024). While we consider this to be a rapid LD decay for chickpea, previous studies have reported LD decay as fast as 0.7 Mbp using a panel of advanced breeding lines and cultivars (Raman et al., 2022). Without having access to a common set of markers, we hypothesize that this is likely due to a different level of genetic diversity. Certainly, rapid LD decay is a common feature of MAGIC populations, such as those created for common bean (Diaz et al., 2020) and cowpea (Huynh et al., 2018), as the successive rounds of crossing followed by multiple generations of SSD assists in recombining linkage blocks, thereby reducing LD (Islam et al., 2016). The fast LD decay is useful to support high‐resolution QTL mapping to reduce the size of the chromosomal interval and reduce the number of candidate genes underlying the QTL, which is important for subsequent QTL introgression, haplotype stacking, or gene discovery studies (Alqudah et al., 2020).

While bi‐parental chickpea populations have been widely adopted in the past to successfully map QTL, this MAGIC population offers advantages and opportunities for future studies. For instance, the allelic variation segregating in the population, and LD decay rate, presents opportunities to map important regions for variety development with multiple alleles at each locus. The large population size of 1135 RILs will also improve the statistical power to detect more and rare QTL, even alleles that are contributed by a single founder, which still occur at a frequency of ∼12.5% in the population.

### 4.3 QTL discovery using the MAGIC population reveals key genomic regions associated with DTF and PHT

In this chickpea MAGIC population, we observed wide phenotypic variation for DTF. Notably, some lines displayed transgressive segregation with earlier and later flowering behavior in comparison to the founders (Figure 8). This phenotypic variation enabled us to map 13 QTLs for DTF across chromosomes 2, 3, 4, 5, 6, and 8. Our analyses reconfirmed flowering as a complex trait, as evidenced by the many loci across the genome, each with relatively small effects. QTL on chromosomes 2 (Q‐DTF2), 4 (Q‐DTF4; Q‐DTF5/Q‐PH1), 5 (Q‐DTF7; Q‐DTF8), and 8 (Q‐DTF11; Q‐DTF12/Q‐PH6; Q‐DTF13) were positioned in close proximity to flowering QTLs reported in previous studies (Abdi et al., 2016; Jaganathan et al., 2015; Kujur et al., 2016; Varshney, Mir et al., 2014). Notably, five QTLs were deemed new, including those positioned on chromosomes 2 (Q‐DTF1), 3 (Q‐DTF1), 6 (Q‐DTF9; Q‐DTF10), and 8 (Q‐DTF13).

Transgressive segregation for PHT was also observed in the MAGIC population. A total of seven QTLs were mapped on chromosomes 4 (Q‐PH1), 5 (Q‐PH2 and Q‐PH3), 6 (Q‐PH4), and 8 (Q‐PH5, Q‐PHT6, and Q‐PH13). Two of the seven QTLs (i.e., Q‐PH1 and Q‐PH6) co‐located with regions detected in previous studies (Gupta et al., 2015; Kujur et al., 2016; Varshney, Mohan et al., 2014). Two QTLs on chromosome 5 (Q‐PH2 and Q‐PH3) were deemed new QTLs for PHT. This highlights the utility of the MAGIC population to discover potentially new loci that could be useful for crop improvement.

Interestingly, we observed a positive correlation between DTF and PHT, suggesting overlapping genetic controls. Our GWAS identified four genomic regions associated with both traits on chromosomes 4 (Q‐DFT5/Q‐PH1), 5 (Q‐DFT7/Q‐DTF8/Q‐PH3), 6 (Q‐DFT9/Q‐PH4), and 8 (Q‐DTF11/Q‐DTF12/Q‐PH6/Q‐PH7/Q‐DTF13) (Table 1). Notably, the same genomic region on chromosome 4 (Q‐DFT5/Q‐PH1) at 12.65–13.06 Mbp co‐located with a QTL reported for vigor (Ca4_Vqtl1) by Nguyen et al. (2022). This suggests the region could be involved in modulating all three traits: vigor, PHT, and flowering time. To confirm this, the MAGIC population could be phenotyped for vigor to test the allele effects on all three traits. Besides, functional categorization analysis of the genes co‐located within ±50 kb of the potential new QTL revealed a strong enrichment of stress‐responsive, developmental, and regulatory genes. Overall, the GWAS results highlight the power of the MAGIC population to dissect the genetic regions underlying complex traits, such as DTF and PHT. The distribution of the detected QTL across chickpea chromosomes indicates that these traits have complex genetic architectures and are both quantitative in nature. Superior MAGIC progeny identified in this study may be useful donor material for inclusion in breeding programs targeting improvement of flowering and PHT, or other important traits of interest.

### 4.4 Haplotype and stacking analyses reveal key allelic combinations for early DTF and taller PHT

Analysis of haplotypes that include multiple markers in high LD can be used to account for the fact that multiple marker alleles are inherited as a unit and are less likely to be affected by recombination (Bevan et al., 2017; Hao et al., 2012). In our study, the highest variance haploblock for DTF was *b000646* on chromosome 8 and *b000250* on chromosome 4 for PHT. Haplotype networks showed genealogies among different haplotypes and graphically display their relatedness. Most importantly, it is constructed and used to reveal the allelic variants that arise from different recombination or chromosomal mutations (Glaszmann et al., 2010). The LD block *b000250* for PHT comprises of 74 makers. The haplotype with the largest effect on PHT at *b000250* was hapPHT7 (Figure 9), which only occurred at a frequency of 1.1% in the population (*n* = 11). The rare haplotype was contributed by founder ICCV 10, yet many other haplotype variants that were more common in the population showed similar mean phenotypes for PHT, for instance, hapPHT4, hapPHT6, and hapPHT5. These desirable haplotypes could assist the selection and breeding of taller chickpea varieties that may be better suited for mechanical harvesting.

Through the in silico stacking analyses, we also demonstrated the potential for combining multiple desirable haplotypes to modulate time to flowering. Genomics‐assisted breeding approaches could be employed using speed breeding methods to generate stacked lines faster (Varshney et al., 2021), which could lead to development of early flowering chickpea, which is a desirable trait in many production enviroments to avoid drought stress and resulting yield losses in chickpea.

## 5 CONCLUSION

This study conducted a genetic characterization of a newly developed MAGIC population derived from eight founders. Our analyses demonstrate the founders, while originally selected for performance in a range of production environments, also represent a high degree of genetic diversity based on co‐analysis of germplasm from Asia and Africa. A large population was developed (1135 lines), and, as anticipated, showed features in common with other MAGIC populations, such as low population structure, high rate of LD decay, and high allelic variation. We highlighted the power of the MAGIC population for trait mapping by studying the genetics of DTF and PHT. Analyses confirmed the role of genomic regions previously reported in the literature and identified several new QTL for both traits. Interestingly, a QTL associated with both DTF and PHT on chromosome 4 (Q‐DTF5/Q‐PH1) co‐located with a QTL reported for vigor by Kujur et al. (2016), suggesting the region may harbor a locus with pleiotropic effects, or alternatively multiple linked loci, that chickpea breeders could potentially exploit to improve all three traits simultaneously. Haplotype and stacking analyses showed the potential for trait improvement through stacking the top 10 haploblocks to develop early flowering chickpea, and selection of desirable haplotypes on chromosome 4 to improve PHT. In summary, the chickpea MAGIC population provides a useful genetic resource for genetics research and pre‐breeding programs for a range of traits important for crop improvement.

## AUTHOR CONTRIBUTIONS

**Oluwaseun J. Akinlade**: Conceptualization; data curation; formal analysis; methodology; writing—original draft. **Hannah Robinson**: Writing—review and editing. **Yichen Kang**: Writing—review and editing. **Mahendar Thudi**: Resources; writing—review and editing. **Srinivasan Samineni**: Resources; writing—review and editing. **Pooran Gaur**: Resources; writing—review and editing. **Millicent R. Smith**: Writing—review and editing. **Kai P. Voss‐Fels**: Writing—review and editing. **Roy Costilla**: Writing—review and editing. **Rajeev K. Varshney**: Resources; supervision; writing—review and editing. **Eric Dinglasan**: Conceptualization; supervision; writing—review and editing. **Lee T. Hickey**: Conceptualization; supervision; writing—review and editing.

## CONFLICT OF INTEREST STATEMENT

The authors declare no conflicts of interest.

## Supporting information



Supplementary Table 1 List and code of parental lines used for the development of the chickpea MAGIC population, including their description and country of cultivation.
Supplementary Table 2. Linkage disequilibrium (LD) among the pairs of markers significantly associated with DTF and PHT to determine those that are linked in order to categorize them into QTL. SNP markers with LD ≥ 0.1 are linked
Supplementary Table 3 Summary statistics of distributions of minor allele frequencies (MAF) and observed heterozygosity in genotypic data of population.
Supplementary Table 4 Summary of the QTL for days to 50% flowering (*Q‐DTF*) and plant height (*Q‐PH*) in the chickpea MAGIC population. Linked markers in LD ≥ 0.1 are highlighted in bold. With the haplo‐blocks co‐located in the same regions.
Supplementary Figure 1. Population structure of the MAGIC population and their founder parents. Principal component analysis based on the pairwise genomic relationship matrix (Roger's distances) calculated from 4255 high‐quality polymorphic SNP markers in 1135 MAGIC lines representing seven crossing designs and eight founder lines. The variation explained by principal components were PC1 = 12.89% and PC2 = 9.97%. The colored triangles represent the parents while the colored circles represent the cross‐design (pedigree).
Supplementary Figure 2 (A) Allele frequency spectra (histogram distributions of minor allele frequency [MAF]). (B) Distribution histograms of observed heterozygosity across the MAGIC population.
Supplementary Figure 3. Genome‐wide profile of the eight MAGIC founder lines. SNP alleles are represented by colors: adenine (A) is indicated in yellow, cytosine (C) is represented in blue, guanine (G) is represented in red and thymine (T) is represented in green.
Supplementary Figure 4. Population structure of the MAGIC population and their founder parents. Principal component analysis based on the pairwise genomic relationship matrix (Roger's distances) calculated from 4255 high‐quality polymorphic SNP markers in 1135 MAGIC lines representing seven crossing designs and eight founder lines. The variation explained by principal components were PC1 = 12.89% and PC2 = 9.97%. The colored triangles represent the parents while the colored circles represent the cross‐design (pedigree).
Supplementary Figure 5. Genome‐wide association mapping for plant height (PHT) and days to 50% flowering (DTF) for 1135 MAGIC lines using 4255 high‐quality SNP markers. *QQ* plots for plant height in blue and days to 50% flowering in green are displayed.

## Data Availability

All data supporting the findings of this study are available within the paper and within its Supporting Information. Genotypic and phenotypic datasets are available from the corresponding author upon request.
