# Supplementary material for: A chickpea MAGIC population to dissect the genetics of complex traits
Source: Plant Genome. 2025 Aug 24;18(3):e70096. doi: 10.1002/tpg2.70096 (PMC12375853; doi:10.1002/tpg2.70096)
Supplement: Supplementary file 1 [file TPG2-18-e70096-s001.pdf]

A chickpea MAGIC population to dissect the genetics of complex traits

Oluwaseun J. Akinlade, Hannah Robinson, Yichen Kang, Mahendar Thudi, Srinivasan Samineni, Pooran Gaur, Millicent R. Smith, Kai P. Voss-Fels, Roy Costilla, Rajeev K. Varshney, Eric Dinglasan, Lee T. Hickey

O. J Akinlade, H. Robinson, Y. Kang, E. Dinglasm, L.T Hickey, Queensland Alliance for Agriculture and Food Innovation, The University of Queensland, Brisbane, Queensland, Australia; M. Thudi, College of Agriculture, Family Sciences and Technology, 1005 State University Dr. Fort Valley State University, Fort Valley 31030, Georgia, United States; M. Thudi, S. Samineni, P. Gaur,International Crops Research Institute for the Semi-Arid Tropics (ICRISAT), Patancheru, Telangana, India; S. Samineni, Crop Diversification and Genetics, International Center for Biosaline Agriculture (ICBA), Dubai, United Arab Emirates; M. R Smith, School of Agriculture and Food Sciences, Faculty of Science, The University of Queensland, Gatton, Queensland, Australia; K.P Voss-Fels, Department of Grapevine Breeding, Hochschule Geisenheim University, Geisenheim, Germany; R. Costilla, Cawthron Institute,, Nelson, New Zealand;O.J Akinlade, R.K Varshney, State Agricultural Biotechnology Centre, Centre for Crop and Food Innovation, Food Futures Institute, Murdoch University, Murdoch, Western Australia, Australia

***Correspondence**

Lee T. Hickey, The University of Queensland, Queensland Alliance for Agriculture and Food Innovation, Brisbane, Australia, l.hickey@uq.edu.au

Eric Dinglasan, The University of Queensland, Queensland Alliance for Agriculture and Food Innovation, Brisbane, Australia, e.dinglasan@uq.edu.au

[OFFICIAL]

Supplementary Table 1: List and code of parental lines used for the development of the chickpea MAGIC population, including their description and country of cultivation.

Founder	Code	Description
ICC 4958	A	Drought tolerant genotype promising for yield in Ethiopia, Kenya, and India
ICCV 10	B	Widely adapted drought-tolerant cultivar found in India and Kenya
JAKI 9218	C	Farmer-preferred cultivar in central and southern India
JG 11	D	Farmer-preferred cultivar in southern India and well-adapted to Kenya
JG 130	E	Farmer-preferred cultivar in central India
JG 16	F	Farmer-preferred cultivar in northern and central India
ICCV 97105	G	Farmer-preferred elite line identified in Kenya and Tanzania
ICCV 00108	H	Farmer-preferred elite line identified in Tanzania

[OFFICIAL]

Supplementary Table 2. Linkage disequilibrium (LD) among the pairs of markers significantly associated with DTF and PHT to determine those that are linked in order to categorise them into QTL. SNP markers with LD ≥ 0.1 are linked.

Chrom	Marker1	Marker1 position	Marker2	Marker2 position	LD
3	M3.16743467	50	M3.23724626	117	0.010085
4	M4.1487970	9	M4.12926479	218	0.013759
4	M4.1487970	9	M4.13421126	231	0.009719
4	M4.1487970	9	M4.17785478	318	0.001759
4	M4.12926479	218	M4.13421126	231	0.856436
4	M4.12926479	218	M4.17785478	318	0.033693
4	M4.13421126	231	M4.17785478	318	0.031513
5	M5.32027655	219	M5.32448226	221	0.035328
5	M5.32027655	219	M5.37601329	289	0.046207
5	M5.32027655	219	M5.38102595	346	0.009044
5	M5.32448226	221	M5.37601329	289	0.001816
5	M5.32448226	221	M5.38102595	346	0.197816
5	M5.37601329	289	M5.38102595	346	0.011811
6	M6.7564965	105	M6.9942982	166	0.135504
6	M6.7564965	105	M6.19084539	386	0.051413
6	M6.9942982	166	M6.19084539	386	0.001919
8	M8.673893	12	M8.2762755	31	0.012744
8	M8.673893	12	M8.3011407	32	0.031368
8	M8.673893	12	M8.4764769	39	0.000388
8	M8.673893	12	M8.5166666	43	3.50E-05
8	M8.2762755	31	M8.3011407	32	0.25623
8	M8.2762755	31	M8.4764769	39	0.036737
8	M8.2762755	31	M8.5166666	43	0.040176
8	M8.3011407	32	M8.4764769	39	0.087833
8	M8.3011407	32	M8.5166666	43	0.155128
8	M8.4764769	39	M8.5166666	43	0.318805

[OFFICIAL]

Supplementary Table 3: Summary statistics of distributions of minor allele frequencies (MAF) and observed heterozygosity in genotypic data of population

Chromosomes	Mean MAF	Mean ObsHet
1	0.319	0.023
2	0.259	0.020
3	0.293	0.019
4	0.242	0.020
5	0.290	0.022
6	0.295	0.021
7	0.319	0.021
8	0.251	0.022
Overall Mean	0.290	0.021

[OFFICIAL]

Supplementary Table 4. Summary of the QTL for days to 50% flowering (*Q-DTF*) and plant height (*Q-PH*) in the chickpea MAGIC population. Linked markers in LD ≥ 0.1 are highlighted in bold. With the haplo-blocks co-located in the same regions.

Q/N	Chr	QTL	Marker.Position	LGEBV BLOCK	DTF -log10(P)	PH -log10(P)	QTL	References
1	2	*Q-DTF1*	M2.32058207	b000143	6.93	NA	Potentially Novel	
2	3	*Q-DTF2*	M3.16743467		5.43	NA	*qtlDtf-3.1*	Abdi et al. (2016)
3	3	*Q-DTF3*	M3.23724626		4.41	NA	Potentially Novel	
4	4	*Q-DTF4*	M4.1487970	b000250	4.03	NA	*qtlDtf-4.1*	Abdi et al. (2016)
5	4	*Q-DTF5*	M4.12926479	b000250	7.37	NA	*qtlDtf-4.2*	Abdi et al. (2016)
	4	*Q-PH1*	M4.13421126	b000250	NA	31.77	*qPH4.1/qtlDtf-4.3, Ca4_Vqtl*	Kujur et al. (2016)
6	4	*Q-DTF6*	M4.17785478	b000256	4.12	NA	*qtlDtf-4.4*	Kujur et al. (2016)
7	5	*Q-PH2*	M5.32027655		NA	4.77	Potentially Novel	
8	5	*Q-DTF7*	M5.32448226		6.34	NA	*qtlDtf-5.1*	Kujur et al. (2016)
	5	*Q-DTF8*	M5.37601329	b000361	5.41	NA	*qtlDtf-5.2*	Kujur et al. (2016)
	5	*Q-PH3*	M5.38102595	b000361	NA	4.07	Potentially Novel	
9	6	*Q-DTF9*	M6.7564965	b000400	6.5	NA	Potentially Novel	
	6	*Q-PH4*	M6.9942982	b000400	NA	4.04	*QR3pht01*	Gupta et al. (2015), Varshney et al. (2014)
10	6	*Q-DTF10*	M6.19084539	b000403	5.73	NA	Potentially Novel	
11	8	*Q-PH5*	M8.673893		NA	4.3	*qPH8.1*	Kujuret al. (2016)
12	8	*Q-DTF11*	M8.2762755	b000627	4.95	NA	*qtlDtf-8.1*	Jaganathan et al. (2015) and Varshney et al. (2014)
	8	*Q-DTF12/Q-PH6*	M8.3011407	b000630	29.96	4.33	*qtlDtf-8.2*	Jaganathan et al. (2015) and Varhney et al. (2014)
	8	*Q-PH7*	M8.4764769	b000633	NA	7.96	*qPH8.1*	Jaganathan et al. (2015), Varhney et al. (2014), Kujuret al. (2016
	8	*Q-DTF13*	M8.5166666		11.15	NA	Potentially Novel	

[OFFICIAL]

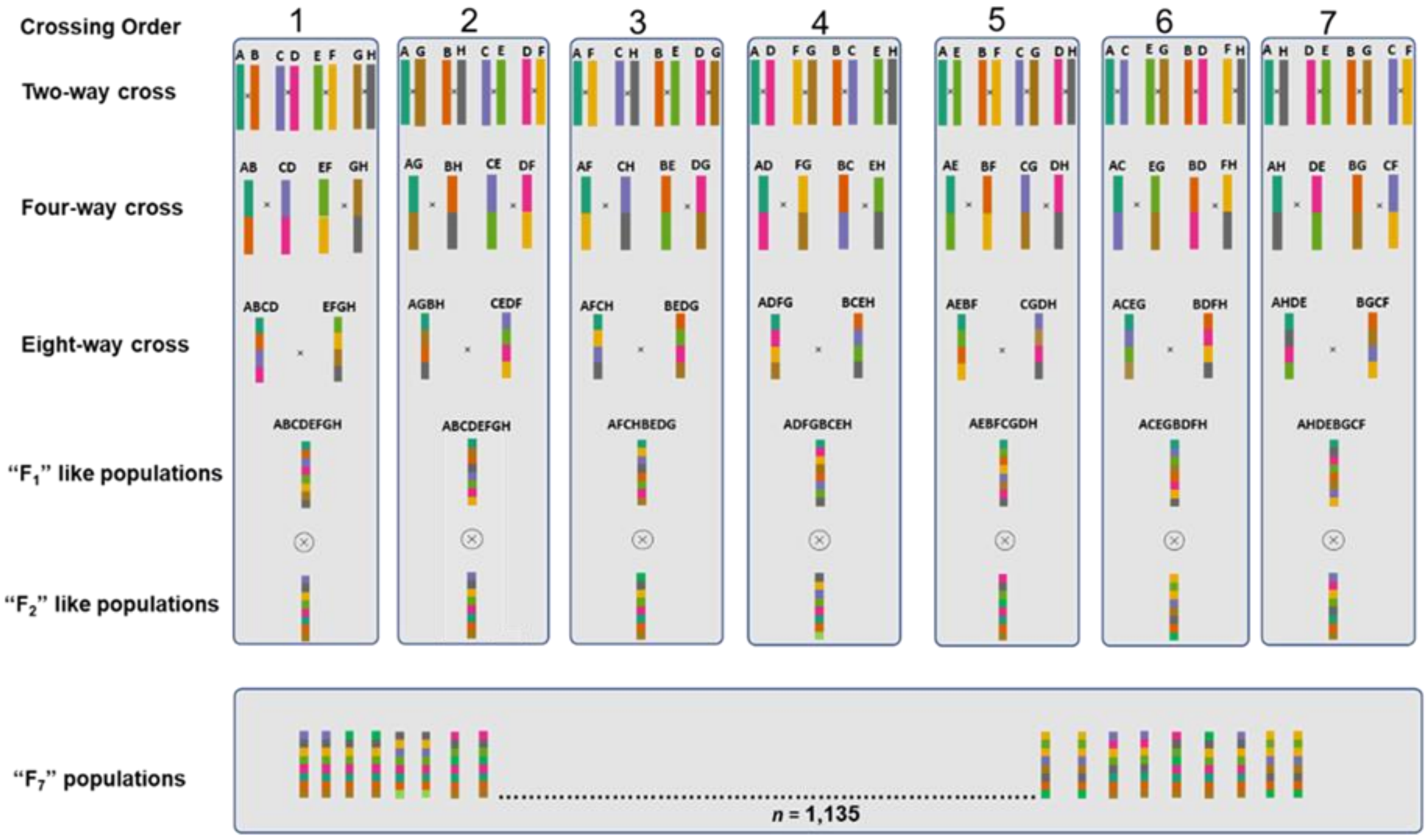

Supplementary Figure 1. Graphical representation of the crossing design and line development for the chickpea MAGIC population. Eight founder lines from Asia and Africa were used to generate 1,135 F7 recombinant inbred lines. Each rectangular box represents the seven families that differ based on crossing order. Each founder is indicated by a distinct colour to demonstrate the contribution and reshuffling of the genome.

[OFFICIAL]

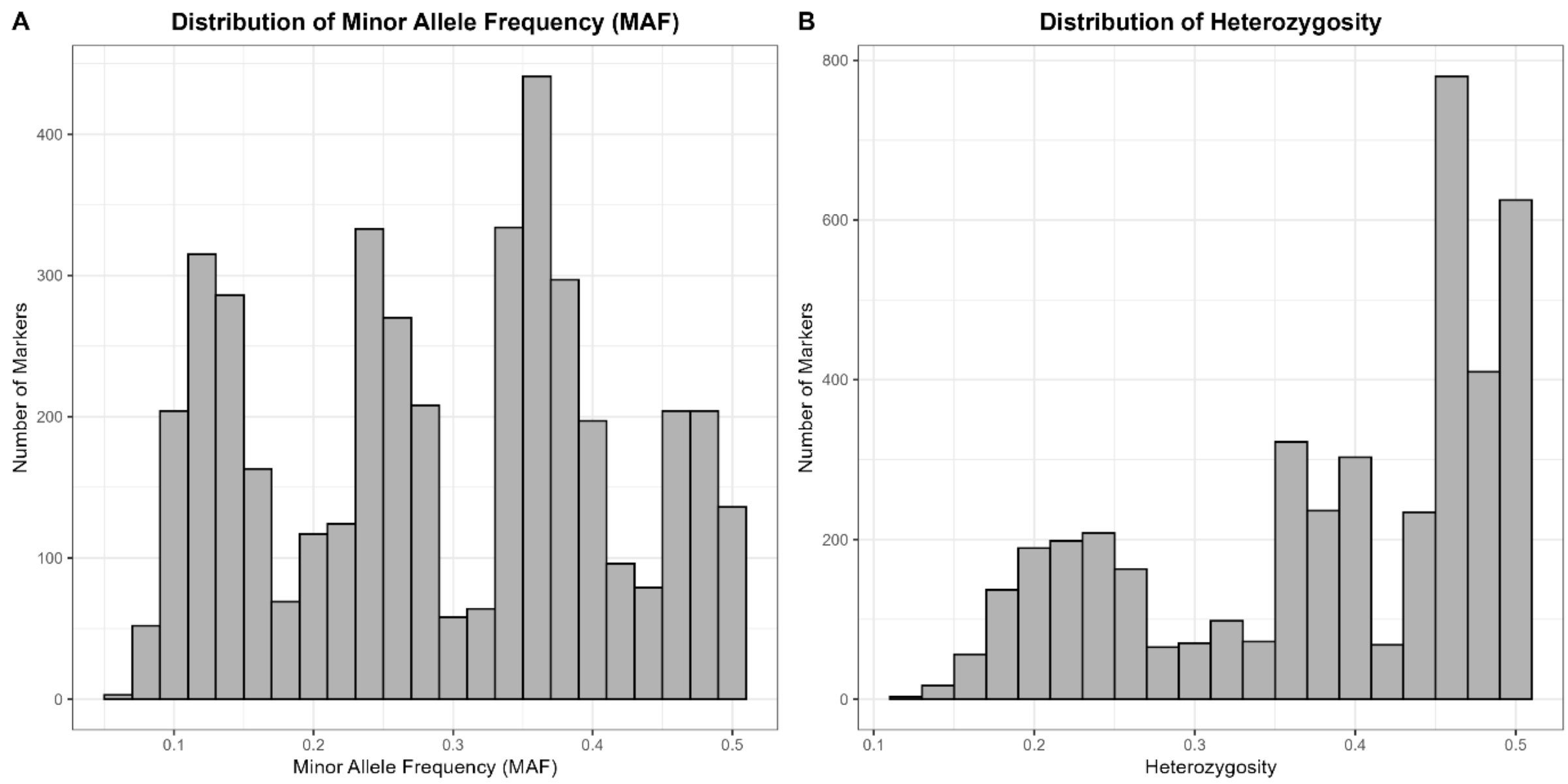

Supplementary Figure 2. (A) Allele frequency spectra (histogram distributions of minor allele frequency (MAF)) (B) Distribution histograms of observed heterozygosity across the MAGIC population.

[OFFICIAL]

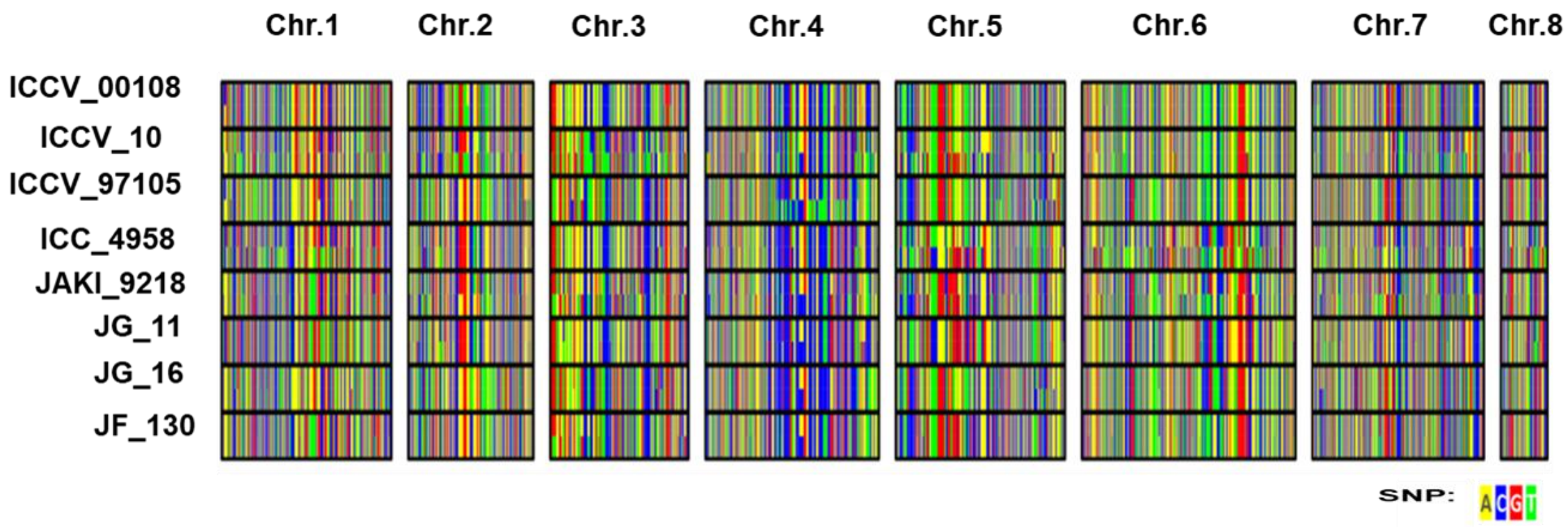

Supplementary Figure 3. Genome-wide profile of the eight MAGIC founder lines. SNP alleles are represented by colours: Adenine (A) is indicated in yellow, cytosine (C) is represented in blue, guanine (G) is represented in red and thymine (T) is represented in green.

[OFFICIAL]

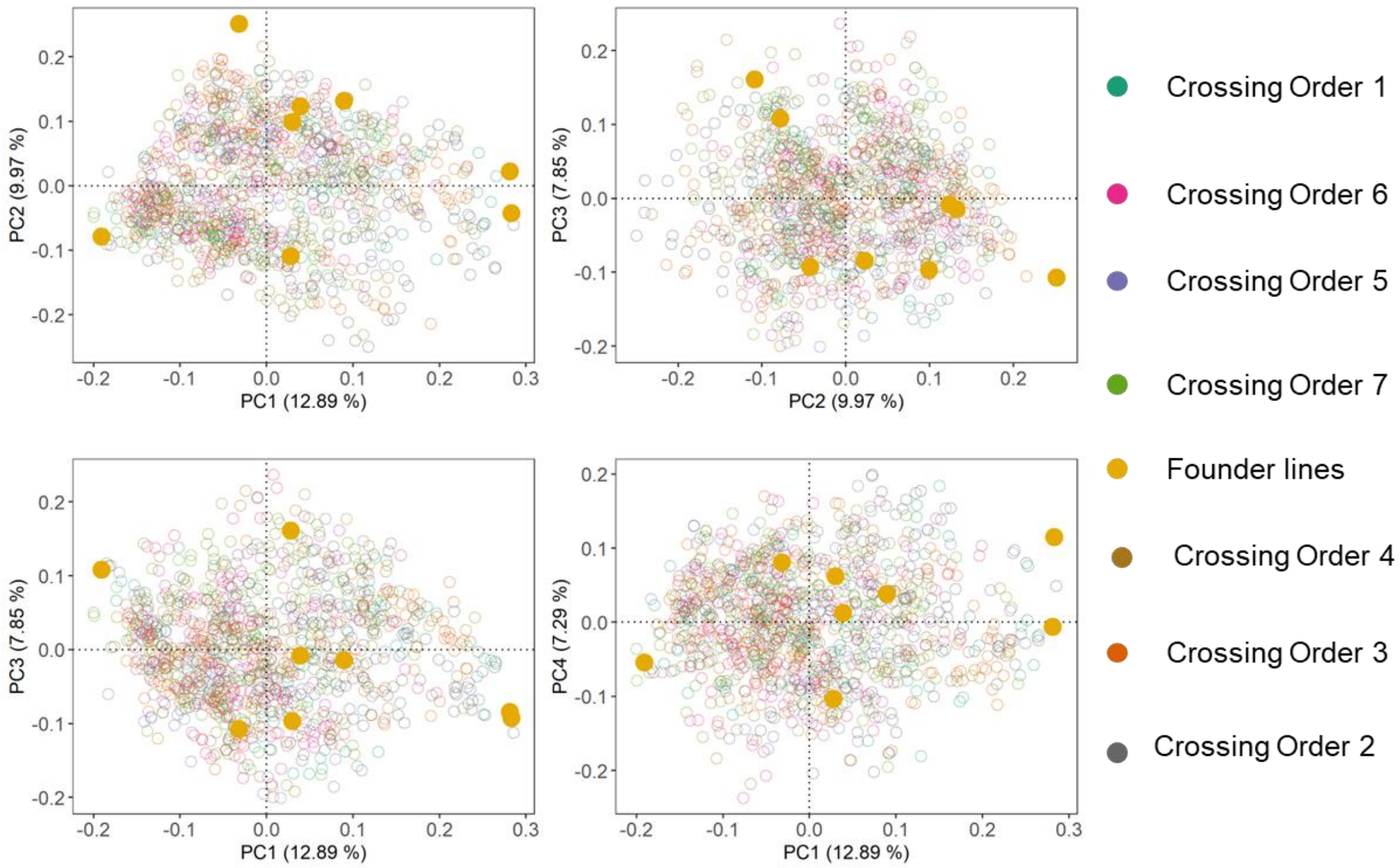

Supplementary Figure 4. Population structure of the MAGIC population and their founder parents. Principal component analysis based on the pairwise genomic relationship matrix (Roger's distances) calculated from 4, 255 high-quality polymorphic SNP markers in 1, 135 MAGIC lines representing seven crossing designs and 8 founder lines. The variation explained by principal components were PC1 =12.89% and PC2 = 9.97%**.** The coloured triangles represent the parents while the coloured circles represent the cross-design (pedigree).

[OFFICIAL]

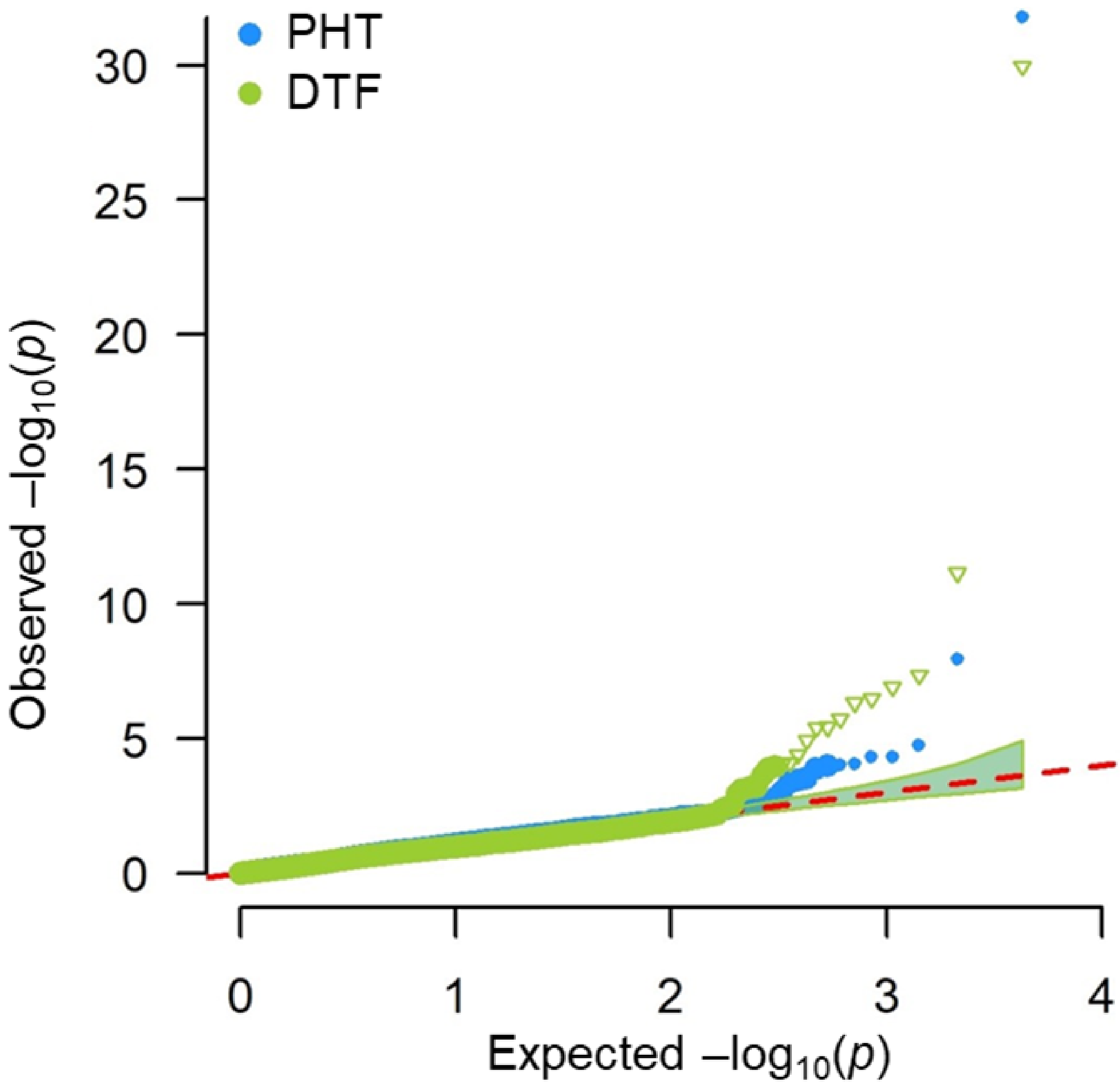

Supplementary Figure 5. Genome-wide association mapping for plant height (PHT) and days-to-50%-flowering (DTF) for 1,135 MAGIC lines using 4,255 high-quality SNP markers. QQ plots for plant height in blue and day-to-50%-flowering in green are displayed.

[OFFICIAL]
